# Medical and Philosophical Intelligence

**Published:** 1817-09

**Authors:** 


### MEDICAL AND PHILOSOPHICAL INTELLIGENCE.

*To the Editors of the London Medical and Physical Journal.*

GENTLEMEN,

I TAKE the liberty of addressing you upon this occasion, in consequence of being unable to obtain the requisite information by any other means.

In the autumn of the year 1814, I was bound to a country apothecary for the term of four years; since that time the Apothecaries' Act has passed, which specifies five years, and that all those who have not served that time cannot be admitted to an examination at Apothecaries' Hall. Now, I have been told, that the Act has a retrospective power, which appears to me to be so very absurd that I cannot credit it. I understood, myself, that Act was only put in force on the 1st of August, 1815; in that case, it does not at all interfere with me, and I suppose I may be admitted to an examination as readily as if I had served an apprenticeship of five years. But, on the contrary, I suppose I must be rebound at the expiration of my present term for another of five years, by which time I shall have attained my twenty-ninth year. If, gentlemen, you will have the goodness to state, in your Journal, whether or not I can be admitted to an examination, you will confer an obligation upon

Your humble servant,

*Chester; 31st of July,* 1817. A STUDENT.

[In answer to our Chester Student, we could remark that all reasonable people are dissatisfied with the clause to which he refers, and we believe the most favourable construction of it is always made. If a gentleman is five years *in a shop*, it is considered sufficient. Probably the clause will be altered whenever the Bill is revised.

We are much obliged to Χειρεργος, and much pleased with his good intentions. But we have always thought it best to avoid the subjects to which he alludes.—Our early motto was "*Quod Medicorum est, Promittant Medici.*" The great extent of our matter is more than enough to occupy our monthly pages. For that reason, we avoided noticing the work of which he tenders us a critique, conceiving it best passed over, as the controversy in which we should have found it necessary to engage could be of no service to our readers.—EDIT.]

---

Since the accounts of extraordinary union by the first intention have appeared in our Journal, we have been favoured with so many others, that our correspondents must allow us to epitomize.

Mr. W. D. ROLFE, surgeon-accoucheur in Bristol, informs us, that in year 1790, he succeeded in restoring the finger and thumb of a shoemaker's apprentice, which was so nearly separated by an axe as to induce the whole neighbourhood to laugh at the attempt.

Dr. BALFOUR, after several compliments for our candour, which we hope are not misplaced, favours us with a letter from Mr. CLOUGH, of South Shields, containing the two following cases:

"The subject of the first was that of a youth, about sixteen years of age, who had one of his great toes struck off entirely, with the exception of a small thread of skin at the under side, but which could be of no manner of service in supporting circulation; I replaced the parts, and in a month re-union was complete.

"The second case was that of a youth also, aged fifteen years, who had his fourth and fifth toes struck off almost entirely, with the addition of an extensive wound above the roots of the toes; the instrument, an axe, which inflicted it, having fallen on the foot in a slanting direction. With my success in the former case before my eyes, and which I candidly avow far exceeded my expectations; I, with the utmost confidence, pursued the same plan of cure in this as in that case, and with the same happy result.

"I have seen both young men frequently since the completion of the cures, and both of them have the same use and command of their toes as they had before they met with the accidents."

We should remark, that in neither of these cases was the separation entire; but Mr. Clough assures his correspondent, that, encouraged by his success, he shall in future make attempts on cases which frequently occur in his neighbourhood, but which he has hitherto thought beyond relief.

We receive the following from Paris, which we acknowledge we should not have admitted, but from the respectability of our correspondent, and inasmuch as the authority on which they rest is unreservedly stated.

*Examples of the Re-union of Parts totally separated from the rest of the Body;* by H. DUTROCHET, D. M. (Translated from La Gazette de Santé of 21st March, 1817.)

*Chareau pres Chateau Regnault;*

SIR, 5 *Mars*, 1817.

The Number of the 1st of March of your interesting Gazette, offers two examples of re-union of parts totally separated from the rest of the body. This physiological phenomenon is at present so well proved, that one may, without exposing oneself to ridicule, so unjustly cast upon Garengeot, relate analogous facts. Those I am about to narrate are almost incredible, but they are attested to me by an ocular witness,—by a man too much above the vulgar to be led away by popular tales,—by General P***, my brother-in-law, who commanded in chief, during a long time, the regular troops of Daoulat-rao-Scindiah, Sovereign of the Mahrattas of Berar.

The amputation of the nose being a very common punishment in India, every means has been had recourse to, to remedy the hideous deformity resulting from it. Two methods are practised in operating the restoration of the nose cut off. The first consists in drawing down a portion of skin from the forehead, which has been employed with so much success by Mr. Carpue, of London. The second method consists in *grafting*, in the place of the nose cut off, with a piece of skin and *tissu cellulaire* subjacent from the parts about the hips.

A sub-officer of cannoneers in the army of General P. was particularly hated by one of his officers, who, profiting by the absence of his general, and of a slight fault committed by the sub-officer, cut his nose off. The mutilated object had recourse to the Indians known for operating the restoration of the nose. This was their method:—The amputation of the nose not being recent, and the wound beginning to cicatrize, they refreshed it, and cut away the edges. They then selected a part of the upper part of the thigh, which they struck repeatedly with an *old shoe*, until they produced a considerable swelling: they then cut off from this part a triangular piece, which they applied to the nose, and affixed it with adhesive plasters. This animal grafting succeeded perfectly, and the man served several years after in General P.'s army.

The following, from the same source, is still more extraordinary. General P., crossing a friendly country, had strictly forbidden all pillage. A man was brought before him taken in the fact. He instantly ordered one of his ears to be cut off. This man was a Brahmin, as are nearly all the writers attached to the Indian armies; and this punishment, being infamous, excited a great tumult,

 which which was kept down by money. In the mean time the question was to replace the ear, which had been thrown away and lost. The ear of a *Paria* was purchased of him: it was cut off, and grafted in the place of the Brahmin's ear, and it succeeded. We may observe, *en passant*, that on this occasion the Brahmins do not appear to have felt the horror that the presence of a Paria ordinarily inspires them with.

Many attempts have been made to operate this grafting on animals. I have myself made several experiments of the kind on rabbits, but without success.

---

*On an additional Test for Arsenic;* By JOHN SPENCER HARRISON, Surgeon, Alstonefield, Staffordshire.

IN cases of poisoning, the country is greatly indebted to Dr. Neale of Exeter, for proving that the tests usually employed for arsenic in the humid way, will produce precipitates similar in appearance to those which might happen where no arsenic had existed upon the stomach. The case now stands as follows:—1st, The contents of a stomach with arsenic, tested by carbonate of potash and nitrate of silver, produce a yellow precipitate. 2ndly, A stomach without arsenic, tested with nitrate of silver, also produces a yellow precipitate, in case it previously contained phosphate of soda, and this last exists in many animal fluids. 3rdly, A stomach with arsenic, tested by carbonate of potash and sulphate of copper, produces a green precipitate. 4thly, A stomach without arsenic, tested with sulphate of copper, would also produce a green precipitate, by adding a small quantity of decoction of onions, and dropping into it a solution of phosphate of soda; which two last might have been present on the stomach.

On conducting these experiments, I find, that, in both the yellow and green precipitates, a very conspicuous distinction may be made by one and the same agent. On adding the mild nitrate of mercury to No. 1, the colour is not altered, but even rendered more permanently yellow. On adding this test to No. 2, it instantly changes the precipitate to a white, which gradually becomes a little grey. This test, added to Nos. 3 and 4, will change both precipitates to a white colour; with No. 3, an effervescence is produced, and the precipitate is somewhat flocculent; while, with No. 4, the precipitate is a more brown white, having a harsh feel. The mild nitrate is prepared by dissolving mercury in diluted nitric acid, keeping an excess of running mercury; it may be made in a phial, having its lower end plunged into cold water.

---

*At a Meeting of the Associated Apothecaries and Surgeon-Apothecaries of England and Wales, held by Public Advertisement, at the Crown and Anchor Tavern, August the 20th,* 1817;

The EIGHTH REPORT of the General Committee was read, as follows:

Circumstances having rendered it expedient to convene a General Meeting of the Members of the Association, its Committee begs leave leave to detail the events which have occurred since the publication of their last Report, dated 24th of April, 1816.

By a reference to that document, it will be seen what steps the committee has taken to procure such alterations as appeared adviseable in the bill intended to be brought before parliament, by the College of Surgeons during that session; and also an amendment of the Apothecaries' Act. The Royal College of Surgeons did not, however, then introduce any bill. But the Court of Assistants of the Society of Apothecaries, notwithstanding the declaration of its bill-committee, "that such practical inconvenience had not arisen from the alledged defects of the act, as to induce it to recommend to the court of assistants any immediate application to parliament," did immediately afterwards intimate, that as the secretary at war, Lord Palmerston, was about to bring in a bill to amend the act, as far as it affected army and navy medical officers, the court meant to embrace an obliging offer of his lordship of making such other alterations in the act as would correct its defects, &c.: and Dr. Burrows and Mr. Field met the bill-committee at the hall, to discuss the points which were stated as requiring amendment. This intention of the society was made known to your committee; and some resolutions relative to the subject were passed on the 15th of May. But the session closed; and the amending bill was not brought forward, either by Lord Palmerston or by the society.

In January last the chairman received a copy of a new bill which the College of Surgeons had arranged. This was submitted to the committee on the 21st of that month. The committee approved this bill, as far as it related to the practice of surgery; but reiterated its objection to the amount of fees for diplomas, as being too large; considering that by the act every person, henceforward practising surgery, would be compelled to apply for a diploma; whereas, under present circumstances, his coming before the college is entirely optional. A request was consequently transmitted to the college, that it would re-consider the subject of fees; accompanied with a copy of the following resolution: viz. "that this committee anxiously hope that the Royal College of Surgeons will not neglect the opportunities allowed by the intended bill of examining into the qualifications of candidates in the knowledge of Midwifery."

This bill was expected to be introduced early in the last session of parliament; but that has also passed without any application being made.

Five years have elapsed since your committee was appointed; during which, it has been reduced by the death of many valuable members, by secessions and other causes, from forty-five to about twelve or fourteen effective persons;—a number, by far too few to perform the ordinary business of the association, or to constitute a due representation of the great body of general practitioners.

The intended resignation of the chairman, Dr. Burrows, is a circumstance which your committee has most sincerely to lament; for in him, from the length of time he has so honourably and zealously laboured in that very ostensible and important situation, all the most material material details of the business have centered. His retirement imposes the obligation of appointing a successor. It would be an act of injustice to Dr. Burrows, if the committee withheld from the association, the fact, that so long ago as May 1816, he expressed, and for very sufficient reasons, his determination to retire from the chair: but under the impression that the duties of the committee would most likely be terminated with the last session of parliament, he acceeded to the general wish of the committee, and was prevailed upon to extend his valuable services till that period. And, although no application has been preferred to parliament, either by the College of Surgeons or by the Society of Apothecaries, his anxiety and exertions have been unremitting to promote, as far as was in his power, the introduction of a bill for the regulation of surgery, and the administration of the Apothecaries' Act agreeably both to its letter and spirit.

By the subjoined statement of the funds of the association, the committee confidently trust it will appear, that a just regard has been paid to so important a resource; and although it may be deemed right to appropriate a portion of it for especial purposes at this meeting, yet it should ever be respected and preserved as a means applicable (when real occasion exists) to the support of the interests of the general practitioners in medicine. Nor can the committee forbear expressing a hope, that the subscription book will still continue open, in order that the fund may be adequate to all possible exigencies.

The remaining members of your committee have thought it a duty incumbent upon them to convene this general meeting of the association; that they may render to it an account of their proceedings; and, with the utmost respect, they here surrender the trust which has been confided to them, and which they have endeavoured to execute faithfully and usefully.

*State of the Fund, May 1, 1817.*

| Dr. | £. | s. | d. |
|---|---|---|---|
| Law expenses - - - | 144 | 9 | 9 |
| Printing - - - - - | 77 | 18 | 7 |
| Advertising - - - - | 56 | 17 | 3 |
| Sundry Disbursements | 43 | 14 | 4 |
| Meetings, Public & Committee, during 4 years. | 24 | 9 | 10 |
| | 347 | 9 | 9 |

| Cr. | £. | s. | d. |
|---|---|---|---|
| Balance of cash in hand at last Audit (Sept. 3, 1813.) | 209 | 14 | 4 |
| Subscriptions since received | 146 | 5 | 1 |
| By Sale of the intended Bill | 7 | 7 | 7 |
| By return on Advertisements | 3 | 13 | 6 |
| By 4 years' interest on Exchequer Bill of £1000 | 201 | 8 | 7 |
| | 568 | 9 | 1 |
| Balance £220 19 4 | | | |
| Exchequer Bill £1000 | | | |

John Hunter, R. S. Wells, } Auditors.

Dr. Burrows having resigned the chair, James Parkinson, Esq. was unanimously elected to fill that situation; after which the following resolutions were passed:

1. Resolved, unanimously, That the most sincere thanks of this association be given to Dr. Burrows for the indefatigable zeal, ability, and disinterestedness, with which he has filled the chair since the formation of the association in 1812: and for the manly perseverance with which he met all the difficulties that were opposed to to the passing of "An Act for the better regulating the Practice of Apothecaries," &c.; by which the honour and respectability of the profession have been upheld, and the public interest most importantly served.

2. Resolved, unanimously, That a purse of five hundred guineas be presented to Dr. Burrows, the late chairman, as an acknowledgment of the services he has rendered this association, to the profession, and to the public.

3. Resolved, unanimously, That the above resolutions be advertised in the public papers.

4. Resolved, unanimously, That the gentlemen who may this day be appointed treasurers to this association be authorized forthwith to carry into effect the two preceding resolutions.

5. Resolved, unanimously, That the sincere thanks of this association be given to the members of the general committee; who, for a period of five years, have, with unwearied zeal, ability, and attention, devoted their valuable time to the interests of the association, and the general improvement of the profession; and who have consequently rendered an essential service to the public.

6. Resolved, unanimously, That the thanks of this association be given to the Treasurers for their services.

7. Resolved, unanimously, That the Report of the general committee, now read, be received and adopted.

8. Resolved, unanimously, That this association do continue permanently.

9. Resolved, unanimously, That this association determines to use its utmost exertions to promote the improvement of those branches of the medical profession exercised by general practitioners, and to protect their interests.

10. Resolved, unanimously, That the interests of the association be entrusted to a committee, with full powers to act according to its discretion.

11. Resolved, unanimously, That the said committee shall consist of twenty-four members, resident in London and its vicinity; and that one moiety of them shall be members of the Society of Apothecaries, and the other moiety of non-members of that society.

12. Resolved, unanimously, That Messrs. Hunter, Upton, Wells, Bowman, De Bruyn, Brande, Cates, Drew, Kerrison, Shillito, Seaton, and Malim, members of the Society of Apothecaries; and that Messrs. Parkinson, Humby, A. T. Thomson, Cook, Haden, James, Reginald Williams, Hayes, Ogle, Neville Wells, and Edward Leese, non-members of the society, do constitute the present committee.

13. Resolved, unanimously, That the present treasurers to this association be desired, and are hereby authorised, to transfer to the treasurers to be appointed at this meeting, the balance of the fund of this association, which may be now in their or the banker's, Messrs. Gosling and Sharpe's hands.

14. Resolved, unanimously, That the moneys belonging to the association be invested in public securities, bearing interest; and be placed in the names of the three treasurers now to be nominated; except such balance as is necessary to defray current expenses.

15. Resolved

15. Resolved, unanimously, That Messrs. Parkinson, Hunter, and Upton, be treasurers; and that the signature of two of the treasurers be necessary to the disposal of any part of the fund.

16. Resolved, unanimously, That in order to establish a competent fund to preserve the interests of the association, further subscriptions be received; and that any new subscriber of one guinea be enrolled as a member of the association.

17. Resolved, unanimously, That subscriptions be received by any of the treasurers, and by the bankers to the association, Messrs. Gosling and Sharpe, No. 19, Fleet-street.

18. Resolved, unanimously, That the committee be authorised by this general meeting to receive from any member of this association any well-authenticated case of illegal practising; for the purpose of submitting the same to the Court of Assistants of the Society of Apothecaries for their prosecution.

19. Resolved, unanimously, That there shall be a general meeting of the association held annually, on the third Wednesday in July, at two o'clock; when any vacancy in the committee, or the office of treasurer, shall be filled up, the accounts of the current year be audited, and all other affairs relating to the association shall be reported.

20. Resolved, unanimously, That a fortnight's notice shall be given, by advertisement in the London Medical Journals, and in two morning and two evening papers, of the time and place of the annual meeting.

The Secretary being under the necessity of resigning—

21. Resolved, unanimously, that the thanks of this meeting with a purse of one hundred guineas, be presented to Mr. Ward, the late secretary, as a mark of estimation for his services.

22. Resolved, unanimously, That the treasurers be requested forthwith to fulfil the object of this resolution.

23. Resolved, unanimously, That Mr. John Powell of Newman-street, be appointed secretary.

24. Resolved, unanimously, That the thanks of this meeting be given to R. M. Kerrison, Esq. for the zeal displayed by him in his publications; as well as for the general interest he has taken to forward the objects of the association.

25. Resolved, That a communication be made from this association to the Court of Assistants of the Society of Apothecaries, begging them to ascertain and publish a list of those persons who were in practice up to the moment of the operation of the act.

26. Resolved, unanimously, That the thanks of this association be given to James Parkinson, Esq. for having accepted the chair; and for the able manner in which he has conducted the business of the meeting.

27. Resolved, unanimously, That the thanks of this meeting to the Chairman be advertised in the public papers.

28. Resolved, unanimously, That the Report of the committee, the state of the funds, and the above resolutions, be sent to the *Medical Journals* for insertion.

JAMES PARKINSON, Chairman.

We

We find the opinion concerning the *suction influence* of the heart, in accounting for the motion of the blood through the veins, is not confined to Liverpool. Dr. J. ZUGENBUHLER, in *Recueil periodique de la Societé de Medecine de Paris,* has published a similar opinion in Latin. However plausible this opinion might be, it seems difficult to reconcile to the condition of those animals which have no lungs and no pulmonary heart. Nor can we well understand how this influence can extend to the circulation through the liver. Dr. Z. has, however, provided for all this, and even for the passage of lymph through its proper vessels.

"Respecting the motion of the lymph, (says he) no plausible explanation has been advanced by physiologists. No vascular elasticity, no muscular action, or arterial vibration can here operate on the innumerable tubes of the absorbent system, and their tenacious lymph; nor will the theory of capillary tubes solve the difficulty in granulated vessels. Suction, then, caused by the vacuum of the heart, demands our consideration; for, by this, the thoracic duct pours its contents, drop by drop, into the left subclavian vein, and thus, at a slow but unerring pace, exhausts the lymph from the whole body. The granulated structure of these vessels resembles the valves of veins; hereby congestion is prevented, and one drop after another is uninterruptedly raised. Nor is it without obvious reason that the thoracic duct empties itself into the left subclavian vein, near the heart; for the left ventricle absorbing the blood more readily from the lungs than the right, may thus exercise a greater power upon the thoracic duct."

We are to reflect, that the "exercise of this power" must extend from the thoracic duct to all the minutest ramifications of lymphatics, and their glands down to the toes. It may be urged, that when a power is once established, it is not easy to ascertain its limits. This we readily grant; but, by the same reasoning, the power of the ventricle assisted by the arteries may be sufficient to convey the blood through the veins, which seems confirmed by an observation of Mr. Hunter, "that in the smaller he perceived a pulsation similar to arterial much more readily than in the larger." Now the former are much nearer the left ventricle, and much farther from the supposed vacuum on the right side of the heart.

An Italian writer has described an hydatid in the human head of the following description:—"It consisted of a head, not unlike that of the tænia, and of a vesicle filled with water and beautifully organized. The vesicle was apparently formed by three different membranes: the first external, delicate, transparent, and shining; under which was observed a series of minute circular fibres; and, lastly, a membrane which invested with its villous surface the interior of the vesicle. Each vesicle, therefore, was but one worm. The cavity of the vesicle contained nothing but water; and not the slightest trace of any organ subservient to the natural functions could be discerned. The figure of the vesicle was round, oblong, or angular. If the animal were alive, on slight pressure of its neck, the head started out, furnished with piercers and a little mouth, resembling those of the *tænia armata*."

 Hydatids

Hydatids in the human brain are so rare, that Dr. Baillie describes none as seen by himself. It is still more curious, that those described in the above paper and in other foreign works, have heads like those found in the brain and other parts of sheep. We trust the Italian work above alluded to, will find its way among us in an English translation, if not, we shall not fail to give an analysis from the original.

---

*Case of Hydrophobia.*—Madame Bruneau, wife of M. Bruneau, of the ordnance department, had her arm violently lacerated by the bite of a cat, about the commencement of November last. The animal fastened upon her with such ferocity, that it would not loosen its hold until some of its bones were broken: it was immediately killed. The laceration was washed with brine, and dressed with some domestic remedy, such as the family had been in the habit of applying to wounds and sores. It continued open for several weeks, and healed at last with much difficulty. About the beginning of May, the scars became inflamed and very itchy, attended with a sort of pinching pain, which extended in the direction of the lymphatics to the axilla, and side of the neck. On the morning of the 12th, when attempting to take a little cordial for the relief of a pain in the stomach, she found herself seized with an indescribable feeling of horror, and constriction of the throat, as the liquid approached her mouth: attributing this to the smell of the cordial, she tried a little tea, and afterwards some water; but the same feeling was excited the instant she looked at either of these. Her husband being employed in the ordnance, sent for a medical officer of that department, who immediately attended, and, after much inquiry, obtained the history above related of the case. Care was taken, in putting the necessary questions to the husband, that the patient should not hear them, in order that she might have no suspicion of the real nature of the disease: she, however, overheard some observations that were made about the cat, and instantly exclaimed, "Ce n'est pas cela, car mon enfant a ete mordu dans le meme tems que moi."

The case being considered an important one, was reported to the Inspector of Hospitals, and permission was obtained from the family to call in an eminent physician, who, upon seeing the case, did not hesitate to coincide in opinion with the ordnance medical officer, that it was a distinct case of hydrophobia. This opinion was, on the following morning, further confirmed by that of the Inspector of Hospitals, and the Surgeon to the Forces. Notice was given of the case to all the medical gentlemen in town who could be found. The progress of the disease was so rapid, as to afford but little time for medical treatment. Copious bleeding having been latterly recommended from high authority, was put in practice, but with evident disadvantage; large doses of mercurial purgatives (indicated by the state of her bowels) were administered with some degree of temporary relief: antispasmodics were then attempted to be given, but the power of deglutition was so soon lost, that very little was taken,

(about three grains of the extract of hyoscyamus). The same sense of horror, and spasmodic constriction of the throat, &c. were excited by looking at the mirror, or any other substance having a polished reflecting surface.

On the morning of the 13th, these sensations came on spontaneously, and were frequently followed by violent convulsions, the moment any liquid was brought in sight. The power of swallowing solids now began to diminish, and, by ten o'clock, not even the saliva could be got down, but issued abundantly out of the mouth in a viscid and stringy state. From this moment the convulsions continued incessantly until two P. M., when she died. The body became perfectly putrid in a few hours after her decease.

*Quebec, May 27.*

---

*List of Patients admitted into all the Civil Hospitals of Paris, from the 1st to the 30th of June, 1817, both inclusive.*

| | |
|---|---|
| Uncharacterised fevers | 25 |
| Intermittent ditto, various | 213 |
| Bilious or gastric ditto | 222 |
| Adynamic or putrid ditto | 19 |
| Catarrhal ditto | 8 |
| Phlegmasiæ, internal and external | 74 |
| Ophthalmia | 37 |
| Rheumatism | 15 |
| Diarrhœa and dysentery | 18 |
| Erysipelas | 4 |
| Phlegmasiæ of the organs of respiration | 99 |
| Phthisis | 44 |
| Apoplexy and recent paralysis | 40 |
| Dropsy and anasarca | 57 |
| Variolous | 5 |
| Metallic colics | 14 |
| Sporadic and chronic disorders, and the result of accidents | 250 |
| Itch | 77 |
| Total | 1205 |

*Meteorological Report.*

| *From April 1 to* | 10 | 20 | 30 |
|---|---|---|---|
| | In. | | |
| Maximum of barometer | 28.4 $\frac{9}{12}$ | 28.5$\frac{6}{12}$ | 28.2$\frac{8}{12}$ |
| Minimum of ditto | 27.11$\frac{3}{12}$ | 27.8$\frac{9}{12}$ | 27.9$\frac{11}{12}$ |
| Max. of thermometer | 22° | 22.6 | 23.5 |
| Min. of ditto | 11°.7 | 8.8 | 14.1 |
| Max. of hygrometer | 91°.5 | 91.0 | 94.0 |
| Min. of ditto | 87.0 | 86. | 82.5 |

---

The following gentlemen have obtained the degree of Doctor in Medicine from the University of Glasgow, within the last twelve months:—Mr. Jas. Yates, from *England;* Messrs. Wm. Couper, Jas. Robertson, James Moore, Daniel M'Allum, Duncan Henderson, John

John Christie, John Macnab, and John M'Dowall, from *Scotland;* Messrs. Francis Jones, Mark Dill, James Campbell, and William Hutton, from *Ireland;* Mr. Matthias Thierens, from *Essequibo;* Mr. John Wood, from *St. Thomas's;* Mr. John Richard Farre Hutson, from *Barbadoes.*

His Royal Highness the Prince Regent has been pleased to appoint WILLIAM WADD, Esq. of Park-place, St. James's, to be Surgeon-Extraordinary to his Royal Highness.

Mr. STEVENSON is appointed Oculist and Aurist to her Royal Highness the Princess Charlotte, and to his Serene Highness the Prince of Saxe-Cobourg.

Dr. ADAMS is elected a Corresponding Member of the Faculté de Medicine (Royal College of Physicians) de Paris.

---

Dr. UWINS, Physician to the City and Caledonian Dispensaries, will commence a Course of Lectures on the Theory and Practice of Medicine, at his house, No. 1, Thavies Inn, Holborn, on the 3d of October, at seven o'clock in the evening; and, in the spring, Dr. Uwins will commence a Course on Materia Medica and Pharmacy.

Dr. ADAMS will commence his Course of Lectures on the Institutes and Practice of Medicine early in the month of October, at his house, No. 17, Hatton Garden.

Dr. CLOUGH, 68, Berners'-street, Physician-Accoucheur to the St. Mary-le-bone General Dispensary, the Endeavour and Benevolent Institutions, &c. announces the re-commencement of his Winter Course of Lectures on the Science and Practice of Midwifery, &c. early in October.

Dr. GEORGE GILBERT CURREY, Fellow of the Royal College of Physicians, and Physician to St. Thomas's Hospital, will commence his Winter Course of Lectures on the Practice and Theory of Medicine, and Materia Medica, on Wednesday, October the 1st.

Dr. CLUTTERBUCK, Physician to the General Dispensary, Aldersgate-street, will begin his Autumn Course of Lectures on the Theory and Practice of Physic, Materia Medica, and Chemistry, on Friday, Oct. 3d, at ten o'clock in the morning, at his house, No. 1, in the Crescent, New Bridge-street. Pupils are admitted, as usual, to attend the medical practice of the Dispensary, where Clinical Lectures on the most interesting cases that occur will be given by the physicians in rotation.

The Lectures on Midwifery, and on the Diseases of Women and Children, by Dr. MERRIMAN, of the Middlesex Hospital, and Dr. LEY, of the Westminster Lying-in Hospital, will be resumed in October next, at the Middlesex Hospital.

Dr. P. M. LATHAM and Dr. SOUTHEY will begin their Lectures upon the Practice of Physic, and the Materia Medica, at the Middlesex Hospital, in the first week of October next.

Mr. CLARKE will commence his Lectures on Midwifery, and the Diseases of Women and Children, on Monday, Oct. 6th, from a quarter-past ten to a quarter-past eleven every morning, at No. 10, Saville-row, Burlington-gardens.

Dr.

Dr. Davis, Physician to the Queen's Lying-in Hospital, and to the Lying-in Charity, will commence his first Winter Course of Lectures on the Theory and Practice of Midwifery, and on the Diseases of Women and Children, on Tuesday the 7th of October, at six o'clock in the evening.

The Medical Lectures in the University of Glasgow will begin on Tuesday, Nov. 4th, at the following hours:—Surgery, by Mr. Burns, at nine in the morning; Dietetics, Materia Medica, and Pharmacy, by Dr. Millar, at ten; Midwifery, by Mr. Towers, at eleven; Practice of Medicine, by Dr. Freer, at twelve; Anatomy, by Dr. Jeffray, at two in the afternoon; Institutions of Medicine, by Dr. Freer, at three; Chemistry and Chemical Pharmacy, by Dr. Cleghorn, at seven.—Dr. Graham will commence his Lectures on Botany about the beginning of May next.

*Anatomical and Medical School of St. Thomas's and Guy's Hospital.*—The usual Lectures given at these Hospitals will commence the beginning of October, viz. at St. Thomas's: Anatomy and Operations of Surgery, by Mr. Astley Cooper and Mr. Henry Cline; Principles and Practice of Surgery, by Mr. Astley Cooper.—At Guy's: Practice of Medicine, by Dr. Curry and Dr. Cholmeley; Chemistry, by Dr. Marcet and Mr. Allen; Experimental Philosophy, by Mr. Allen; Theory of Medicine, and Materia Medica, by Dr. Curry and Dr. Cholmeley; Midwifery, and Diseases of Women and Children, by Dr. Haighton; Physiology, or Laws of the Animal Œconomy, by ditto; Structure and Diseases of the Teeth, by Mr. Fox.

*Medical School, St. Bartholomew's Hospital.*—The following Courses of Lectures will be delivered at this Hospital during the ensuing winter, to commence October 1st:—On the Theory and Practice of Medicine, by Dr. Hue; on Anatomy and Physiology, by Mr. Abernethy; on the Theory and Practice of Surgery, by ditto; on Chemistry and Materia Medica, by Dr. Hue; on Midwifery, by Dr. Gooch; Practical Anatomy, with Demonstrations, by Mr. Stanley.

*London Hospital.*—Lectures on the following subjects will be given at this Hospital, to commence in October:—Anatomy and Physiology, by Mr. Headington; Surgery, by ditto; Midwifery, by Dr. Ramsbottom; Chemistry, by Mr. R. Phillips; Materia Medica and Pharmacy, by ditto.—Mr. R. Phillips will commence a Course of twenty-four Lectures on Chemistry at No. 66, Cheapside, on the 6th of October, at seven in the evening. *(See our Cover.)*

*Great Windmill-street.*—Mr. Wilson and Mr. Bell will commence their Lectures on Anatomy and Surgery on the 1st of October, at two o'clock. Mr. Bell will deliver a separate Course of Lectures on Surgery in the evening. The Anatomical Demonstrations will be given by Mr. Shaw, who will direct the students in their dissections. —A Clinical Lecture will be given every week in the Middlesex Hospital.

*Theatre of Anatomy, Hatton Garden.*—Lectures on Anatomy, Physiology, Pathology, and Surgery, by Mr. J. Taunton, Member of the Royal College of Surgeons, &c. will commence on Saturday,

October 4th, at eight o'clock in the evening precisely; and be continued every Tuesday, Thursday, and Saturday, at the same hour.

Mr. GOOD, F.R.S. will commence his Course of Lectures on Nosology, Medical Nomenclature, and the Theory and Practice of Medicine, on Monday, Sept. 29th, at the Crown and Rolls Rooms, Chancery-lane. The course will rather exceed three months, and be repeated three times a-year. From the comprehensiveness of the subject, a Lecture will be given daily, instead of every other day, as is the common practice. The introductory Lecture will commence at half-past three o'clock in the afternoon; the subsequent Lectures at eight in the morning.

Mr. CURTIS, Aurist to his Royal Highness the Prince Regent, and Surgeon to the Royal Dispensary for the Diseases of the Ear, will commence his next Course of Lectures on the Anatomy, Physiology, and Pathology of the Ear, on Wednesday, October the 1st, at seven o'clock in the evening, at the Royal Dispensary, Carlisle-street, Soho-square.

Mr. BROOKES will commence his Autumnal Course of Lectures on Anatomy, Physiology, and Surgery, on Wednesday, October 1st, at the Theatre of Anatomy, Blenheim-street. *(See our Cover.)*

---

Dr. JOHN MAYO proposes to publish some Remarks on Insanity, in addition to those already published by Dr. Thomas Mayo.

Mr. RICHARD WILLIAMS has in the press a work entitled "An Analysis of the Medicinal Waters in Llandrindod, in Radnorshire;" and dedicated (by permission) to her Royal Highness the Princess Charlotte.

In the press, and in six weeks will be published, in one closely printed volume, octavo, about 300 pages, An Essay on the Prolongation of Life, and Conservation of Health; unfolding original views and fundamental principles for their attainment; and embracing observations on the nature cause, and treatment, of some of the principal diseases which assail the British constitution in its native climate. To which is added an Appendix, containing practical researches on the pathology, treatment, and prevention of Gout, in all its proteian forms: translated and condensed from the French of M. M. Guilbert and Hallé, (just published,) with notes. By JAMES JOHNSON, M.D. Surgeon to his Royal Highness the Duke of Clarence, author of the Influence of Tropical Climates on European Constitutions, and one of the editors of the Medico-Chirurgical Journal and Review.

In the press, and will be published on the 1st of October next, A Catalogue of an extensive Collection of Books in Anatomy, Medicine, Surgery, Midwifery, Chemistry, Botany, &c. new and second-hand, sold by Anderson and Chase, Medical Booksellers, West Smithfield: to which is added, a complete List of the Lectures delivered in London, with their terms, hours of attendance, &c. with Tables of the Pay of the Medical Department of the Army, Navy, and East-India Company's service, &c.

Dr. ADAMS is preparing a new edition of his Life of the late Mr. J. Hunter.

---

### A METEOROLOGICAL JOURNAL,

By Messrs. WILLIAM HARRIS and Co. 50, Holborn, London.

*From the 20th July to the 19th August, inclusive.*

| Day of Month. | Rain gauge | Therm. | | | Barom. | | De Luc's Hygrom. Dry | | Damp | | Wind. | | Atmospheric Variation. | | |
|---|---|---|---|---|---|---|---|---|---|---|---|---|---|---|---|
| July | | | | | | | | | | | | | | | |
| 20 | ,10 | 65 | 67 | 60 | $29^9$ | $29^9$ | | | 3 | 3 | W | SW | Fine | Rain | Fine |
| 21 | ,07 | 64 | 68 | 60 | $29^8$ | $29^8$ | | | 2 | 2 | S | S | Fine | | |
| 22 | ,04 | 63 | 69 | 59 | $29^8$ | $29^8$ | | | 3 | 3 | SW | SW | Rain | Fine | |
| 23 | 05 | 60 | 69 | 57 | $29^7$ | $29^7$ | | | 3 | 5 | W | NW | Fine | Rain | Fine |
| 24 | ,12 | 59 | 67 | 58 | $29^8$ | $29^8$ | | | 3 | 4 | NW | W | Fine | Rain | Fine |
| 25 | ,06 | 60 | 68 | 57 | $29^9$ | $29^9$ | | | 3 | 3 | SW | W | Rain | Clo. | Fine |
| 26 | ,10 | 61 | 69 | 57 | $29^9$ | $29^6$ | | | 5 | 3 | S | SW | Rain | | Clo. |
| 27 | ,07 | 60 | 67 | 54 | $29^6$ | $29^7$ | | | 2 | 2 | SW | SW | Rain | Fine | |
| 28 | ,14 | 60 | 65 | 55 | $29^8$ | $29^9$ | | | 2 | 3 | W | SW | Fine | Rain | |
| 29 | ,16 | 62 | 66 | 57 | 30 | $29^8$ | | | 3 | 3 | SW va. | S | Fine | Clo. | Rain |
| 30 | ,19 | 61 | 66 | 53 | $29^7$ | $29^7$ | | | 3 | 3 | SW | SW | Fine | Rain | |
| 31 | ,10 | 57 | 67 | 52 | $29^7$ | $29^7$ | | | 2 | 2 | SW | SW | Fine | Rain | Fine |
| August | | | | | | | | | | | | | | | |
| 1 | ,05 | 57 | 65 | 54 | $29^8$ | $29^9$ | | | 2 | 2 | WSW | SW | Fine | stormy | Fine |
| 2 | ,07 | 58 | 64 | 52 | $29^9$ | $29^7$ | | | 2 | 2 | WSW | W | Fine | | Clo. |
| 3 | | 56 | 66 | 54 | $29^7$ | $29^8$ | | | 2 | 2 | SW | SW | Fine | | Clo. |
| 4 | | 56 | 65 | 55 | $29^9$ | $29^9$ | | | 1 | 1 | SW | W | Fine | | Clo. |
| 5 | ,09 | 59 | 65 | 54 | $29^9$ | $29^9$ | 0 | 0 | 0 | 0 | S | E | Fine | | |
| 6 | | 59 | 70 | 57 | 30 | 30 | 0 | 0 | 0 | 0 | SW | SW | Fine | | |
| 7 | | 63 | 69 | 59 | $29^8$ | $29^9$ | 0 | 0 | 0 | 0 | SE | SE | Fog | Fine | Rain |
| 8 | ,07 | 63 | 69 | 59 | $29^9$ | $29^7$ | | | 1 | 1 | SW | S | Rain | | Clo. |
| 9 | 04 | 63 | 67 | 58 | $29^7$ | $29^6$ | | | 1 | 1 | SW | S | Fine | Rain | |
| 10 | ,13 | 64 | 67 | 57 | $29^5$ | $29^4$ | | | 2 | 2 | S | SEE | Fine | Rain | |
| 11 | ,10 | 67 | 67 | 57 | $29^4$ | $29^4$ | | | 2 | 3 | SW | SW | Fine | Rain | |
| 12 | ,09 | 69 | 69 | 61 | $29^3$ | $29^4$ | | | 3 | 3 | SW | WSW | Fine | Clo. | Rain |
| 13 | ,07 | 66 | 69 | 59 | $29^4$ | $29^4$ | | | 3 | 3 | SW | SSW | Fog | Clo. | Rain |
| 14 | 06 | 66 | 66 | 59 | $29^5$ | $29^6$ | | | 3 | 4 | SW | SSW | Fog | Clo. | Rain |
| 15 | ,09 | 66 | 66 | 60 | $29^6$ | $29^6$ | | | 3 | 4 | S | S | Rain | | |
| 16 | ,13 | 65 | 65 | 60 | $29^8$ | $29^7$ | | | 4 | 4 | S | S | Rain | | |
| 17 | ,11 | 64 | 67 | 59 | $29^7$ | $29^5$ | | | 5 | 5 | SW | SE | Fine | | |
| 18 | | 66 | 65 | 58 | $29^7$ | $29^7$ | | | 7 | 7 | S | SW | Fine | | Rain |
| 19 | ,06 | 63 | 65 | 58 | $29^7$ | $29^6$ | | | 5 | 5 | S | SW | Rain | Clo. | |

The quantity of rain fallen in the month of July is 1 inch and 85-100ths.

REPORT

### REPORT OF DISEASES.

The town has been free from any serious epidemic for a long while. The latter end of the month has, however, been visited with an autumnal diarrhœa, which, in a few instances, has shown a tendency to dysentery, and, in one case, with such marks of inflammation as to require the lancet.

Three old subjects have gone through all the stages of *febris lenta nervosa,* with slight delirium and symptoms somewhat formidable, had they been accompanied by others to encrease the alarm. Two had singultus for three days with very little intermission, and it continued after the delirium had ceased and the tongue had become clean. The third had apthæ so alarming as to prevent deglutition for several days, and to produce a fœtor which seemed to indicate deeper mischief. They all recovered; but, in the last case, the fever was protracted to twenty-one days. The season has been less favourable to children, whether affected with the exanthemata dentition or hooping-cough.

